# Supplementary material for: Spatial Patterns of Endometriosis Incidence. A Study in Friuli Venezia Giulia (Italy) in the Period 2004–2017
Source: Int J Environ Res Public Health. 2021 Jul 5;18(13):7175. doi: 10.3390/ijerph18137175 (PMC8297028; doi:10.3390/ijerph18137175)
Supplement: Supplementary file 1 [file ijerph-18-07175-s001.zip › Supplementary File S1-proofed.pdf]

Supplementary File S1: SENSITIVITY ANALYSIS

Part 1: Comparing areas at higher/lower risk

To compare areas at higher and lower risk within the Region, we used the Chi-square test for categorical variables, and the non-parametric Wilcoxon Mann Whitney test for ordinal or continuous variables.

Table S1 shows a comparison between the case of endometriosis identified in the 5 high risk municipalities and those identified in the other areas of the Region, by identification source, and the few demographic variables available from the data sources used. Overall, women resident in the 5 municipalities had a higher frequency of histological diagnosis of endometriosis (73.6% vs 53.8%, $p < 0.0001$) and a higher age at diagnosis (41.0 yrs old on average vs 37.0, $p < 0.0001$). No difference was seen concerning the place of birth.

Table S1: Comparison between endometriosis cases identified in the 5 high risk municipalities and those identified in the other areas of the Region.

	**5 high risk municipalities**<br>**N = 296**	**Rest of the region**<br>**N = 3829**	**p-value**
Type of identification, n (%)			<0.0001
Pathology	128 (43.2%)	680 (17.8%)	
Hospitalization	78 (26.4%)	1768 (46.2%)	
Hospitalization and Pathology	90 (30.4%)	1381 (36.0%)	
Age at diagnosis, median (IQR) [a]	41.0 (32.5–45.0)	37.0 (31.0–43.0)	0.0001
Born outside Italy, n (%)	44 (14.9%)	545 (14.2%)	0.76

[a] IQR = Interquartile Range

Part 2 Capture-recapture analysis

We found five municipalities with higher risk with regard to the regional average.

We studied the distribution of cases by the two identification sources in the five municipalities and in the rest of FVG region. We aimed to evaluated potential

ascertainment bias which could explain the risk difference between the five municipalities and the rest of the FVG Region, using capture-recapture analysis. [S1-1]

The table below reports the frequency of cases by identification source.

**High Risk Area**		
	Pathology	
Hospital Discharge	Yes	No
Yes	a=90	b=78
No	c=128	d=NA

**Other FVG Region areas**		
	Pathology	
Hospital Discharge	Yes	No
Yes	a=1381	b=1768
No	c=680	d=NA

Summary statistics on these data are:

Agreement:

High Risk Area

Conditional probability of rating positive given rated positive p_s= 0.47

Cohen's Kappa on p_s= - 0.0497

Other FVG Region areas

Conditional probability of rating positive given rated positive p_s= 0.53

Cohen's Kappa on p_s= 0.0995

Conditional probabilities

High Risk Areas

P(HD+|P+)= 0.41

P(P+|HD+)= 0.54

Other FVG Region areas

P(HD+|P+)= 0.67

P(P+|HD+)= 0.44

There is evidence for lack of agreement between the two sources and lower probability of being reported by HD given positive reporting by Pathology in the 5 high risk municipalities. These data suggest a tendency to report accidental endometriosis findings in Pathology reports where HD records did not mention endometriosis as principal diagnosis.

Capture-recapture analysis is used to estimates coverage. The estimator is a function of the association odds ratio (OR) between the two identification sources, Hospital Discharge and Pathology. Coverage is defined as the number of identified cases divided by the total number of cases. The total number of cases is equal to the number of identified cases plus the number of missing cases. The estimate for the number of missing data is d=(bc/a)*OR (see the table above) and can be estimated from the observed data assuming a value for the Odds Ratio (OR). The confidence interval for the number of missing cases when OR=1 is obtained from a log-linear model with reverse coding in order to have the constant term denoting the expected count for the missing cell; for OR different from 1, the confidence interval comes from a log-linear prediction using log(OR) as offset. [S1-2]

	**High Risk Area**		**Other FVG Region areas**	
OR_a	Missing cases	Coverage	Missing cases	Coverage
0.5	55<br>(95% CI 39; 79)	84.3%<br>(95% CI 78.9; 88.4)	435<br>(95% CI393; 482)	89.8%<br>(95% CI 88.8; 90.7)
1	111<br>(95% CI 78; 157)	72.7%<br>(95% CI 65.3; 79.1)	870<br>(95% CI 785; 965)	81.5%<br>(95% CI 79.9; 83.0)
2	222<br>(95% CI 156; 315)	57.1%<br>(95% CI 48.4; 65.5)	1741<br>(95%CI 1571;1930)	68.7%<br>(95% CI 66.5; 70.9
2.5	277<br>(95% CI 196; 393)	51.6%<br>(95% CI 43.0; 60.2)	2176<br>(95%CI 1963;2412)	63.8%<br>(95% CI 61.4; 66.1)

As said before, we found evidence of a lack of agreement between HD and P sources, the conditional probabilities are close to 0.50. Assuming an association OR=1, we estimate, for Other FVG Region areas, a coverage of 82% (95% CI 80; 83). To have similar coverage in the High Risk Area of the 5 municipalities we should assume an association OR lesser then one. (see table above)

In other word the relationship between the two sources of ascertainment in the High Risk Area is negative fixing to one that in the other FVG Region areas. Or, symmetrically, fixing the association OR to one for the High Risk Area we should assume an association OR of 1.65.

The relative risk of endometriosis observed in the areas with regard to the regional average is 1.5.

Fixing a coverage equal to that of the High Risk Areas under the assumption of an association OR of one, we should assume for the other FVG Region areas an association OR = 1.65, and as a consequence we have to increment the number of cases for the other FVG Region areas of 563. Indeed, the greater number of endometriosis by Pathology only in the High Risk Areas is suggestive of an ascertainment bias and the capture-recapture analysis estimates a deficit of 563 cases for the other areas of FVG Region. Adding these cases, the relative risk of the High Risk Area will change from 1.5 to 1.31 (95% CI 1.13; 1.52). The limits of the confidence interval are the lower limit of the supported range when we use the lower relative risk form the confidence interval of the missing cases and the opposite for the upper limit.

In conclusion, adjusting for ascertainment bias the observed cluster of cases in the High Risk Area was maintained.

Other more complex analysis would be to map incidence using two imperfect measures, hospital discharge and pathology, by bivariate Bayesian shared model. This however assumes that we cannot rely on an Endometriosis Register and would be more appropriate to map prevalence. A second more important problem is that sensibility ad specificity of the two imperfect measures are spatially varying, a problem not easy to model in that framework. We could include coverage probabilities as a covariate in a Bayesian mapping, and in case by hierarchical modelling to propagate uncertainty. Estimates of coverage probabilities could be very extremely unstable at municipality level and some identifiability problem could arise unless more complex hierarchical model be specified. The amount of empirical information on coverage is weak.

REFERENCE

S1-1. Chao A, Tsay PK, Lin SH, Shau WY, Chao DY. The applications of capture-recapture models to epidemiological data. *Stat Med* 2001;20(20):3123-57. doi: 10.1002/sim.996 [published Online First: October 3, 2001].

S1-2. Baillargeon S, Rivest LP. Rcapture: Loglinear Models for Capture-Recapture in R. *Journal of Statistical Software* 2007;19(5). doi:10.18637/jss.v019.i05 [published Online First: April 3, 2007].
