# Supplementary material for: Spatial Patterns of Endometriosis Incidence. A Study in Friuli Venezia Giulia (Italy) in the Period 2004–2017
Source: Int J Environ Res Public Health. 2021 Jul 5;18(13):7175. doi: 10.3390/ijerph18137175 (PMC8297028; doi:10.3390/ijerph18137175)
Supplement: Supplementary file 1 [file ijerph-18-07175-s001.zip › Supplementary File S2-proofed.pdf]

Supplementary File S2: Appendix to Table 2

Table S2. Distribution of endometriosis cases and population by age classes.

Municipality	Number of cases age 15-20	Number of cases age 21-25	Number of cases age 26-30	Number of cases age 31-35	Number of cases age 36-40	Number of cases age 41-45	Number of cases age 46-50	Women residing in the municipality * age 15-20	Women residing in the municipality * age 21-25	Women residing in the municipality * age 26-30	Women residing in the municipality * age 31-35	Women residing in the municipality * age 36-40	Women residing in the municipality * age 41-45	Women residing in the municipality * age 46-50
San Canzian d'Isonzo	0	4	3	7	7	13	4	142,21	121,50	153,57	202,93	250,21	266,43	241,00
Staranzano	1	1	3	9	5	9	13	193,07	141,79	154,79	209,43	272,57	302,71	294,07
Ronchi dei Legionari	0	3	14	12	8	15	10	292,50	242,79	275,36	380,50	481,50	525,79	470,79
Monfalcone	1	2	9	20	15	39	28	608,07	576,57	676,29	814,71	917,79	987,71	923,36
Grado	1	2	6	6	8	10	8	169,57	168,00	191,14	226,43	269,57	309,57	329,21
Lusevera	0	2	1	2	0	1	0	12,00	12,29	15,21	18,64	20,36	25,07	24,43
San Lorenzo Isontino	0	0	4	2	0	0	5	28,43	23,71	38,36	50,57	62,79	65,93	59,29
Fiumicello	0	3	3	2	5	8	5	106,21	96,07	127,93	169,57	189,57	183,21	178,50
Codroipo	1	5	6	16	16	16	12	335,93	343,29	459,07	554,29	593,64	616,29	576,57
Mariano del Friuli	0	0	1	2	0	4	3	36,29	27,00	37,14	52,36	57,36	58,57	50,14
Morsano al Tagliamento	0	0	2	2	4	5	2	58,64	59,14	72,21	83,43	96,00	98,14	100,07
Latisana	1	2	16	20	9	10	6	325,29	294,71	378,43	480,07	550,93	577,00	543,36
Gradisca d'Isonzo	0	2	2	4	7	5	11	156,93	123,00	142,71	191,14	238,71	275,29	272,93
Capriva del Friuli	0	0	2	3	3	2	1	40,93	33,64	41,29	56,21	70,50	77,29	71,93
Turriaco	1	1	1	5	2	2	4	58,79	45,86	67,14	94,50	117,86	124,79	108,29
Prata di Pordenone	1	6	9	6	2	9	9	209,50	216,29	275,21	323,21	344,93	326,93	284,79
Barcis	0	0	0	0	0	2	0	3,36	4,21	4,36	4,14	4,79	9,36	10,64
Polcenigo	0	1	2	2	1	5	6	66,00	55,21	76,36	105,07	130,43	132,57	121,07
Cordenons	1	6	9	15	17	19	11	470,57	416,00	469,21	563,57	691,14	770,71	748,07
Fiume Veneto	1	2	9	11	10	9	11	289,57	250,36	320,14	409,00	467,29	485,86	436,43
Arba	1	0	2	1	2	1	1	27,71	27,36	40,64	40,93	48,36	49,50	47,07

Dolegna del Collio	0	0	0	1	1	0	1	8,79	7,79	8,86	8,71	12,71	19,07	16,93
Mossa	0	1	1	3	0	3	1	36,50	30,36	37,21	42,79	59,57	71,00	67,79

*Numbers represent the sum of women residing in the municipality in the fourteen years considered.
