# Supplementary material for: Spatial Patterns of Endometriosis Incidence. A Study in Friuli Venezia Giulia (Italy) in the Period 2004–2017
Source: Int J Environ Res Public Health. 2021 Jul 5;18(13):7175. doi: 10.3390/ijerph18137175 (PMC8297028; doi:10.3390/ijerph18137175)
Supplement: Supplementary file 1 [file ijerph-18-07175-s001.zip › Supplementary File S3-proofed.pdf]

Supplementary File S3: Brief description of the high risk area

The high-risk area for endometriosis is a non-rural flatland with an intermediate degree of urbanization. In the Monfalcone catchment area, there are industrial activities, such as manufacturing and construction activities, in particular ship building, and a coal-fired power plant whose spill-over area corresponds for the most part to the area of increased incidence. Monfalcone, in fact, hosts a port of moderate importance, thanks to the availability of large flat areas close to the quays and to good connections with the road, motorway and railway networks, both national and international. Ronchi dei Legionari relies on the presence of the airport and of the connected Intermodal Hub, an integration platform between the different transport systems (air-rail-road). The municipality is also very close to a high-traffic road, the SS14, with an average daily traffic (ADT) of just under 13,000 vehicles, exclusively light type. The area of interest is also characterized by the presence of natural reserves such as 'Valle Cavanata' and the 'Foce dell'Isonzo - Isola della Cona'. The resident population of the 5 high-risk municipalities is just over 62,000 people (5% of the total population of Friuli Venezia Giulia, 50% women), approximately 6% of which is foreign, with a peak 26% in Monfalcone. The population density ranges from 67.4 in Grado to 1395 in Monfalcone, and the average income is around 20,000 euros. [S3-1] The climate is characterized by generally harsh winters and hot summers. The thermoregulatory function of the Adriatic Sea is very limited due to the scarce depth of the waters; the Adriatic, on the other hand, is an important area of convergence and sorting of the air masses from the Atlantic, the Mediterranean and central-eastern Europe. The area is in fact characterized by the Bora, a cold, dry and strong wind with gusts reaching 150 kilometres per hour causing a flow of cold air from the hinterland towards the sea. Another frequent wind is the Scirocco, a hot and humid wind from the South-East that can give rise to violent storm surges on the coast, although the most damaging are the ones caused by the south-westerly Libeccio that encounters no obstacles in its path. The area is also subject to abundant rainfall that reaches 1000 mm per year. [S3-2]

REFERENCE

S3-1. Regione in cifre - https://www.regione.fvg.it/rafvg/cms/RAFVG/GEN/statistica/FOGLIA3/

S3-2. Piano regionale di miglioramento della qualità dell'aria - Legge regionale 16/2007 - http://www.regione.fvg.it/rafvg/cms/RAFVG/ambiente-territorio/pianificazione-gestione-territorio/FOGLIA201/
